# Transcriptome Analysis of the Brown Planthopper *Nilaparvata lugens*

**DOI:** 10.1371/journal.pone.0014233

**Published:** 2010-12-06

**Authors:** Jian Xue, Yan-Yuan Bao, Bao-ling Li, Yan-Bing Cheng, Zhi-Yu Peng, Hang Liu, Hai-Jun Xu, Zeng-Rong Zhu, Yong-Gen Lou, Jia-An Cheng, Chuan-Xi Zhang

**Affiliations:** 1 Key Laboratory of Molecular Biology of Crop Pathogens and Insects of Ministry of Agriculture, State Key Laboratory of Rice Biology, Institute of Insect Science, Zhejiang University, Hangzhou, China; 2 Beijing Genomics Institute-Shenzhen, Shenzhen, China; Massachusetts Institute of Technology, United States of America

## Abstract

**Background:**

The brown planthopper (BPH) *Nilaparvata lugens* (Stål) is one of the most serious insect pests of rice in Asia. However, little is known about the mechanisms responsible for the development, wing dimorphism and sex difference in this species. Genomic information for BPH is currently unavailable, and, therefore, transcriptome and expression profiling data for this species are needed as an important resource to better understand the biological mechanisms of BPH.

**Methodology/Principal Findings:**

In this study, we performed *de novo* transcriptome assembly and gene expression analysis using short-read sequencing technology (Illumina) combined with a tag-based digital gene expression (DGE) system. The transcriptome analysis assembles the gene information for different developmental stages, sexes and wing forms of BPH. In addition, we constructed six DGE libraries: eggs, second instar nymphs, fifth instar nymphs, brachypterous female adults, macropterous female adults and macropterous male adults. Illumina sequencing revealed 85,526 unigenes, including 13,102 clusters and 72,424 singletons. Transcriptome sequences larger than 350 bp were subjected to Gene Orthology (GO) and KEGG Orthology (KO) annotations. To analyze the DGE profiling, we mainly compared the gene expression variations between eggs and second instar nymphs; second and fifth instar nymphs; fifth instar nymphs and three types of adults; brachypterous and macropterous female adults as well as macropterous female and male adults. Thousands of genes showed significantly different expression levels based on the various comparisons. And we randomly selected some genes to confirm their altered expression levels by quantitative real-time PCR (qRT-PCR).

**Conclusions/Significance:**

The obtained BPH transcriptome and DGE profiling data provide comprehensive gene expression information at the transcriptional level that could facilitate our understanding of the molecular mechanisms from various physiological aspects including development, wing dimorphism and sex difference in BPH.

## Introduction

The brown planthopper (BPH) *Nilaparvata lugens* (Stål) is one of the most serious insect pests of rice in Asia. BPH mainly sucks rice phloem sap and transmits plant viruses, *i.e*., the rice ragged stunt virus (RRSV) and the rice grassystunt virus (RGSV) [1]. In China, BPH has caused rice damage amounting to millions of tons per year since 2005 [2]. At present, chemical control remains the first choice technique for BPH management. However, the overuse of insecticides leads to insecticide resistance and planthopper resurgence, and it aggravates environmental pollution [3]. In fact, BPH has been the most problematic plague among insect pests that threaten food and ecological safety of worldwide.

In adult stage, BPH displays two wing forms: long (macropterous) and short (brachypterous). Macropterous adults fly long distances and invade rice-growing areas, whereas brachypterous adults are adapted for reproduction and produce numerous offspring in rice fields [4]. BPH wing dimorphism is affected by environmental factors and population densities [5]. These biological properties are closely related to their distribution, reproduction and ability to cause damage in rice plants.

BPH has been extensively studied from ecological and physiological perspectives, but the molecular regulation mechanisms are poorly understood. At present, the genomic resources available for BPH are scarce. Noda *et al.* (2008) identified more than 37,000 expressed sequence tags (ESTs) in various BPH tissues and stages [4]. Together with the nucleotide sequences obtained by NCBI searches, 37,312 BPH ESTs are available. These data provide useful information for transcriptional, proteomic, and gene functional analysis of BPH. Nevertheless, these genetic data are insufficient for elucidating the molecular mechanism of BPH in agricultural ecological systems.

In recent years, next-generation high-throughput DNA sequencing techniques have provided fascinating opportunities in the life sciences and dramatically improved the efficiency and speed of gene discovery [6]. For example, Illumina sequencing technology offers millions of sequence reads from a single instrument run. This capacity permits gene expression profiling experiments with an improved dynamic range and considerable cost savings. Our approach comes from serial analysis of gene expression (SAGE) [7], which generates 21 bp tags from the 3′ ends of transcripts.

In this study, we generated over three billion bases of high-quality DNA sequence using Illumina technology and demonstrated the suitability of short-read sequencing for the *de novo* assembly and annotation of genes expressed in a eukaryote without prior genome information. In this study, we finally obtained 85,526 unigenes of BPH transcriptome. Furthermore, we constructed six digital gene expression (DGE) libraries and compared the gene expression profiles of BPH among different developmental stages, sexes and wing forms. The assembled, annotated transcriptome sequences and gene expression profiles provide useful information for the identification of genes involved in BPH development modulation, wing dimorphism and sex difference.

## Methods

### Insects

The BPH strain was originally collected from a rice field located in the Huajiachi Campus of Zhejiang University, Hangzhou, China. The insects used in this experiment were the offspring of a single female and were reared at 28±0.5°C on rice seedlings (Xiushui 128) under a 12∶12 h light:dark photoperiod. After BPH laying eggs into rice plant for three days, we carefully teared the leaf sheaths using fine-pointed forceps (0.1 mm). About ten eggs could be collected from each leaf sheath. A total of 300 eggs were used in this study. First to fifth instar nymphs can be discriminated by appearance and body size. Nymphs and adults of the same sex and phenotype were collected into a glass tube using an aspirator, respectively. The fresh samples were used to extract total RNA immediately.

### cDNA library preparation and Illumina sequencing for transcriptome analysis

Total RNA was extracted using the SV Total RNA Isolation System (Promega) according to the manufacturer's protocol. To obtain complete gene expression information, a pooled RNA sample including different developmental stages, sexes and wing forms [eggs, 2nd instar nymphs, 5th instar nymphs, brachypterous female adults (BFA), macropterous female adults (MFA) and macropterous male adults (MMA)] was used for transcriptome analysis. All samples of the adult BPH were from four days after adult emergence. According to the Illumina manufacturer's instructions, poly(A)^+^ RNA was purified from 20 µg of pooled total RNA using oligo(dT) magnetic beads and fragmented into short sequences in the presence of divalent cations at 94°C for 5 min. The cleaved poly(A)^+^ RNA was transcribed, and then second-strand cDNA synthesis was performed. After the end-repair and ligation of adaptors, the products were amplified by PCR and purified using the QIAquick PCR Purification Kit to create a cDNA library.

The cDNA library was sequenced on the Illumina sequencing platform (GAII). The raw reads from the images were generated using Solexa GA pipeline 1.6. After removal of low quality reads, processed reads with an identity value of 95% and a coverage length of 100 bp were assembled using SOAP denovo software and clustered using TGI Clustering tools [8], [9]. All raw transcriptome data has been deposited in SRA (NCBI).

The generated unigenes larger than 350 bp were analyzed by searching the GenBank database with the BLASTX algorithm (http://www.ncbi.nlm.nih.gov/). Gene Orthology (GO) and KEGG Orthology (KO) annotations of the unigenes were determined using Blast2go (http://www.blast2go.org/) and InterProScan software.

### Digital gene expression (DGE) library preparation and sequencing

Total RNA was extracted either from eggs, 2nd instar nymphs, 5th instar nymphs, BFA, MFA or MMA using the SV Total RNA Isolation System (Promega). DEG libraries were prepared using the Illumina gene expression sample prep kit. Briefly, poly(A)^+^ RNA was purified from 6 µg of total RNA using oligo(dT) magnetic beads. Double-stranded cDNAs were directly synthesized on the poly(A)^+^ RNA-bound beads and then digested with *NlaIII*. The fragmentized cDNAs containing 3′ ends were purified from the magnetic beads, and then the Illumina adaptor 1 was added to the 5′ ends of these cDNA fragments. After digestion with *MmeI*, an enzyme that recognizes the junction of the Illumina adaptor 1 (sense: 5′ACACTCTTTCCCTACACGACGCTCTTCCGATC3′) and the CATG site, 21 bp tags containing the adaptor 1 sequence were produced. Subsequently, the Illumina adaptor 2 (sense: 5′GATCGGAAGAGCGGTTCAGCAGGAATGCCGAG3′) was ligated to the 3′ ends of the tags to create a tag library. The library was amplified by PCR for 15 cycles, and 85 bp strips were purified from 6% PAGE gels. The single-stranded molecules were attached to the Illumina sequencing chip for sequencing. During this process, adaptor 1 was used as sequencing primer. Each tunnel of chip (flow cell) generated millions of raw tags with a length of 35 bp. All raw tag data have been deposited in SRA (NCBI).

### Analysis and mapping of DGE tags

To map the DGE tags, the sequenced raw data were filtered to remove low quality tags (tags with unknown nucleotide “N”), empty tags (no tag sequence between the adaptors) and tags with only one copy number (which might result from sequencing errors). For tags annotation, the clean tags containing CATG and 21 bp tag sequences were mapped to our transcriptome reference database, allowing no more than one nucleotide mismatch. The clean tags were designated as unambiguous clean tags. For gene expression analysis, the number of unambiguous clean tags for each gene was calculated and normalized to TPM (number of transcripts per million clean tags).

### Evaluation of DGE libraries

To compare the differences in gene expression, the tag frequency in each DGE library was statistically analyzed according to the method described by Audic and Claverie [10]. The false discovery rate (FDR) was used to determine the threshold P-value in multiple tests. A FDR < 0.001 and an absolute value of the log2 ratio >1 were used as the threshold to determine significant differences in gene expression. The differentially expressed genes were used for GO and KO enrichment analyses. Enriched P-values were calculated according to the hypergeometric test:
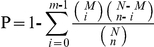



In this equation, N represents the number of genes with GO/KO annotation, n represents the number of differentially expressed genes in N, M represents the number of genes in each GO/KO term, m represents the number of differentially expressed gene in each GO/KO term. For GO enrichment analysis, all of the P-values were performed with Bonferroni correction. We selected a corrected P-value <0.05 as a threshold to determine significant enrichment of the gene sets. In contrast, for KO enrichment analysis, we used a FDR <0.05 as the threshold to determine significant enrichment of the gene sets.

### Quantitative real-time PCR (qRT-PCR) validation

Total RNA was extracted as described for the DGE library preparation and sequencing. The concentration of each RNA sample was adjusted to 1 µg/µl with nuclease-free water, and 2 µg of total RNA was reverse transcribed in a 20 µl reaction system using the AMV RNA PCR Kit (TaKaRa). The sequences of the specific primer sets are listed in Table S1. The 18S rRNA gene of BPH was used as an internal gene. The qRT-PCR was performed using the SYBR Premix Ex Taq Kit (TaKaRa) according to the manufacturer's protocol. The results were normalized to the expression level of the constitutive 18S rRNA gene. A no template control (NTC) sample (nuclease free water) was included in the experiment to detect contamination and to determine the degree of dimer formation (data not shown). A relative quantitative method (ΔΔCt) was used to evaluate the quantitative variation.

## Results

### Illumina sequencing and sequence assembly

**Figure 1:**
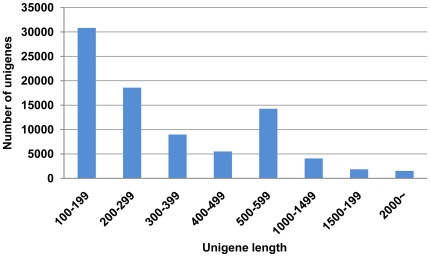
Unigene size distribution. All of the unigene sizes were calculated.

A total of 36,945,096 reads (accumulated length of 2,770,882,200 bp; SRA accession number SRX023419) were generated through Illumina sequencing and assembled into 1,921,675 contigs. Using paired end-joining and gap-filling, these contigs were further assembled into 98,710 scaffolds with a mean length of 295 bp. After clustering the scaffolds together with the nucleotide sequences available at NCBI, we finally obtained 85,526 unigenes, including 13,102 clusters and 72, 424 singletons, with a mean length of 434 bp. The size distribution indicated that the lengths of the 7,405 unigenes were more than 1000 bp (Fig. 1). To ensure precision in mapping the tags to unigenes, unigenes containing a length of more than 350 bp were used as the reference sequences (30,987 unigenes). To evaluate assemble accuracy, we sequenced 200 full length cDNA clones. We searched these cDNA sequences in the 30,987 unigene database using BLASTN with a cut-off E-value of 10^−10^. The results showed a mean identity of 98.5% and query coverage of 76.5%, suggesting that the assembled 30,987 unigenes have a high reliability and cover most of the transcriptome sequences.

### Annotation of predicted proteins

**Figure 2:**
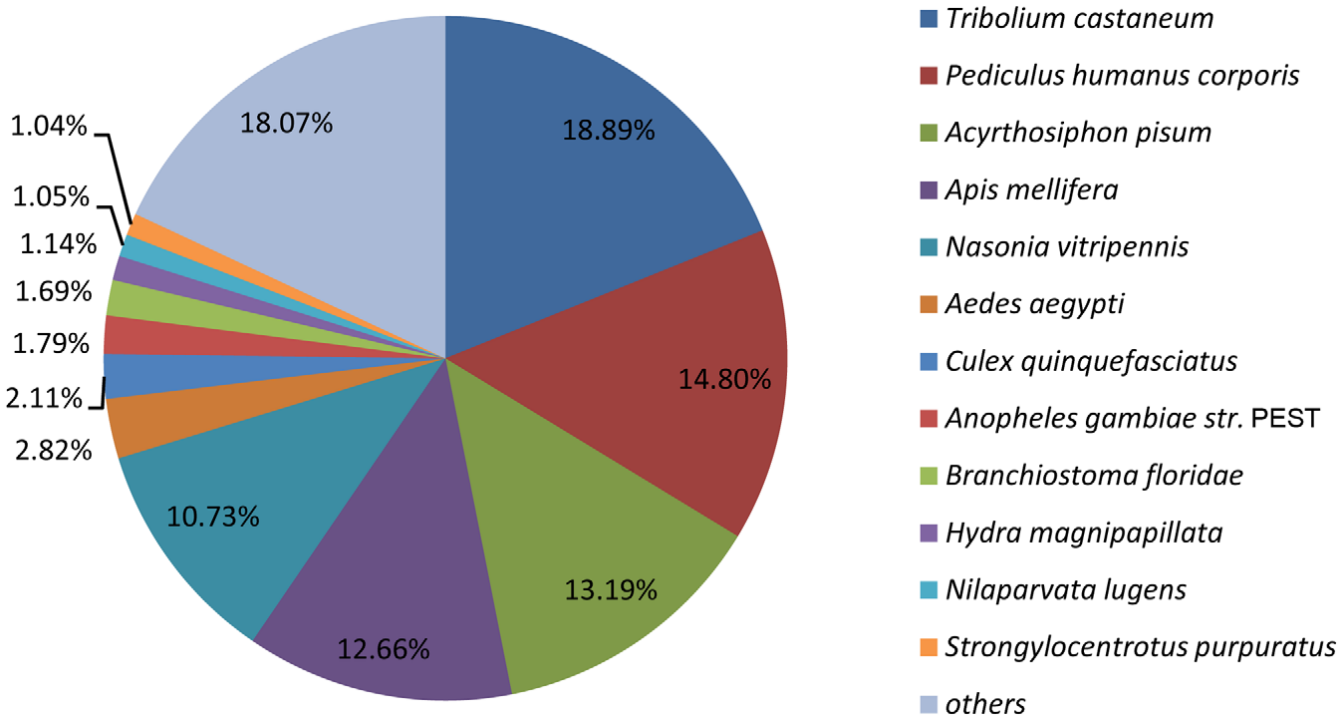
Species distribution of the BLASTX results. This figure shows the species distribution of unigene BLASTX results against the nr protein database with a cutoff E value <10^−5^ and the proportions of each species. Different colors represent different species. Species with proportions of more than 1% are shown.

To annotate these unigenes, we first searched the reference sequences using BLASTX against the non-redundant (nr) NCBI protein database with a cut-off E-value of 10^−5^. A total of 17,388 (56.1% of all distinct sequences) unigenes provided a BLAST result (Table S2). The species distribution of the best match result for each sequence is shown in Fig. 2. The BPH sequences showed 18.89% matches with *Tribolium castaneum* sequences followed by *Pediculus humanus corporis* (14.80%), *Acyrthosiphon pisum* (13.19%), *Apis mellifera* (12.66%) and *Nasonia vitripennis* (10.73%).

### Gene ontology (GO) and Kyoto Encyclopedia of Genes and Genomes (KEGG) ontology (KO) classification

**Figure 3:**
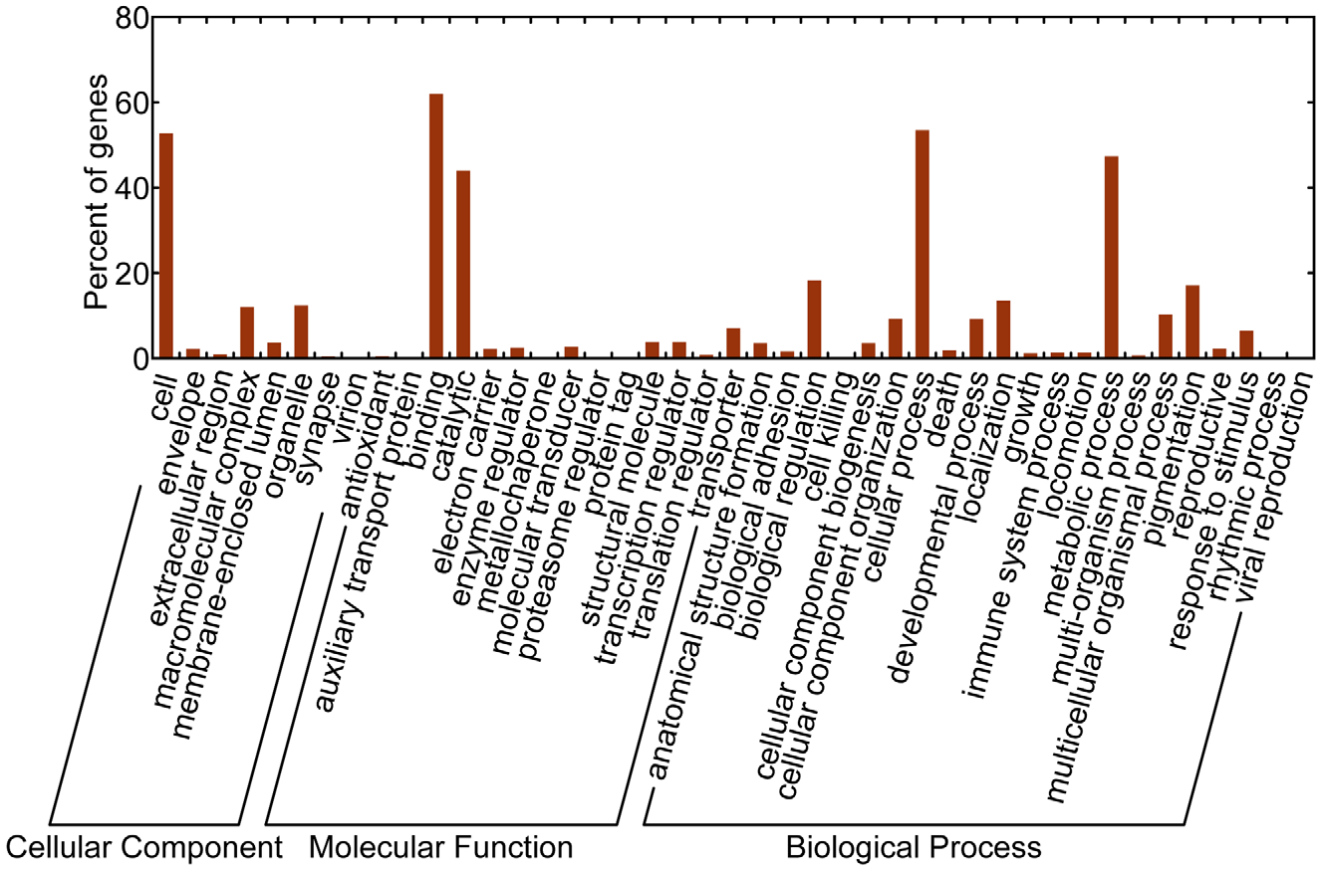
GO categories of the unigenes. Unigenes were annotated in three categories: cellular components, molecular functions, biological processes.

We used GO and KO assignments to classify the functions of the predicted BPH unigenes. Among 30,987 unigenes, approximately 35.0% and 34.7% of the unigenes could be annotated in GO and KO based on sequence homologies, respectively (Table S3 and Table S4). In each of the three main categories (biological process, cellular component and molecular function) of the GO classification, the terms ‘cell’, ‘binding and catalytic’ and ‘metabolic process and cellular process’ were dominant, respectively (Fig. 3).

### DGE library sequencing

**Figure 4:**
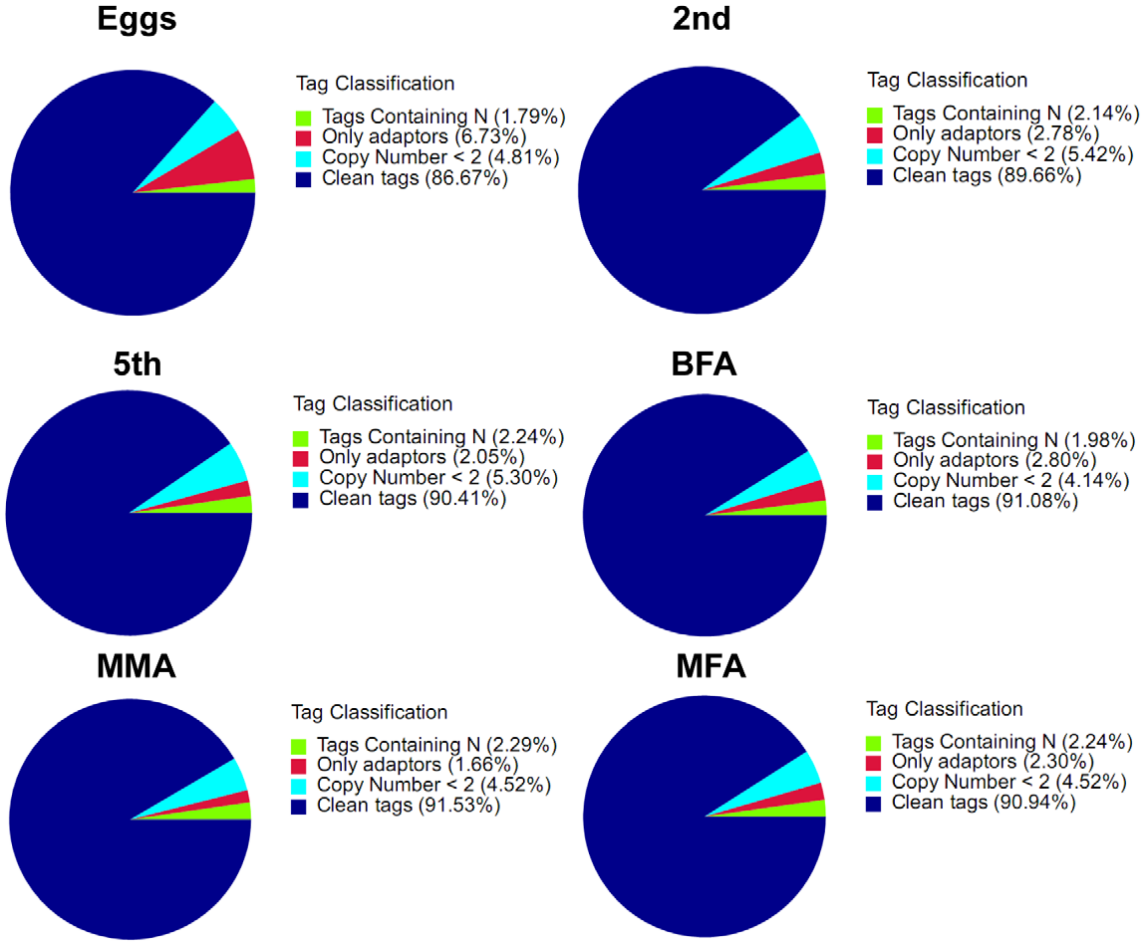
Different components of the raw tags in each sample. The percentages of tags containing N, adaptors, a tag copy number <2, clean tags and raw tags. The numbers in parentheses indicate the percentage of each type of tag among the total raw tags.

Six DGE libraries of BPH were sequenced: eggs (SRX023492), 2nd instar nymphs (SRX023493), 5th instar nymphs (SRX023494), BFA (SRX023495), MFA (SRX023496) and MMA (SRX023497), which generated approximately three million raw tags in each library. After filtering the low quality tags, the total number of clean tags in each library ranged from 2.4 to 3.2 million (Table 1), and the percentage of clean tags among the raw tags in each library ranged from 86.67 to 91.53% (Fig. 4). Among the clean tags, the number of sequences that could be mapped to unigenes ranged from 1.6 to 2.5 million, and the percentage of these clean tags ranged from 67.32 to 84.47% in six libraries (Table 1).

**Table 1:** Tag analysis statistics.

Summary	Eggs	2nd instar nymphs	5th instar nymphs	MFA	MMA	BFA	Averages
Raw Data	2,810,544	3,594,091	3,405,088	3,058,037	3,073,636	3,303,619	3,207,503
Distinct Raw Data	293,115	396,372	369,707	279,132	279,790	274,795	315,485
Clean Tag	2,435,875	3,222,342	3,078,546	2,781,015	2,813,334	3,009,091	2,890,034
Distinct Clean Tag	90,579	126,338	120,545	88,037	89,158	84,022	99,780
Clean Tag/Raw Tag	86.67%	89.66%	90.41%	90.94%	91.53%	91.08%	90.05%
All Tag Mapping to Gene	1,639,815	2,372,365	2,393,681	2,281,666	2,286,082	2,541,902	2,252,585
All Tag Mapping to Gene*	67.32%	73.62%	77.75%	82.04%	81.26%	84.47%	77.74%
Distinct All Tag Mapping to Gene	38,976	60,573	64,253	47,807	47,568	45,766	50,824
Distinct All Tag Mapping to Gene*	43.03%	47.95%	53.30%	54.30%	53.35%	54.47%	51.07%
Unambiguous Tag Mapping to Gene	970,647	1,566,933	1,559,493	1,325,789	1,203,732	1,580,957	1,367,925
Unambiguous Tag Mapping to Gene*	39.85%	48.63%	50.66%	47.67%	42.79%	52.54%	47.02%
Distinct Unambiguous Tag Mapping to Gene	33,345	52,229	55,345	40,499	40,452	38,524	43,399
Distinct Unambiguous Tag Mapping to Gene *	36.81%	41.34%	45.91%	46.00%	45.37%	45.85%	43.55%
All Tag-mapped Genes	13,010	16,744	18,256	14,671	15,269	14,184	15,356
All Tag-mapped Genes**	41.99%	54.04%	58.92%	47.35%	49.28%	45.77%	49.56%
Unambiguous Tag-mapped Genes	11,032	14,654	16,008	12,567	13,118	12,023	13,234
Unambiguous Tag-mapped Genes**	35.60%	47.29%	51.66%	40.56%	42.33%	38.80%	42.71%
Unknown Tag	796,060	849,977	684,865	499,349	527,252	467,189	637,449
Unknown Tag*	32.68%	26.38%	22.25%	17.96%	18.74%	15.53%	22.26%
Distinct Unknown Tag	51,603	65,765	56,292	40,230	41,590	38,256	48,956
Distinct Unknown Tag*	56.97%	52.05%	46.70%	45.70%	46.65%	45.53%	48.93%

Notes:

*% of Clean Tag;

**% of Ref Unigenes.

Statistics of raw tags, clean tags, tags mapped to unigenes, unambiguous tags and unknown tags.

**Figure 5:**
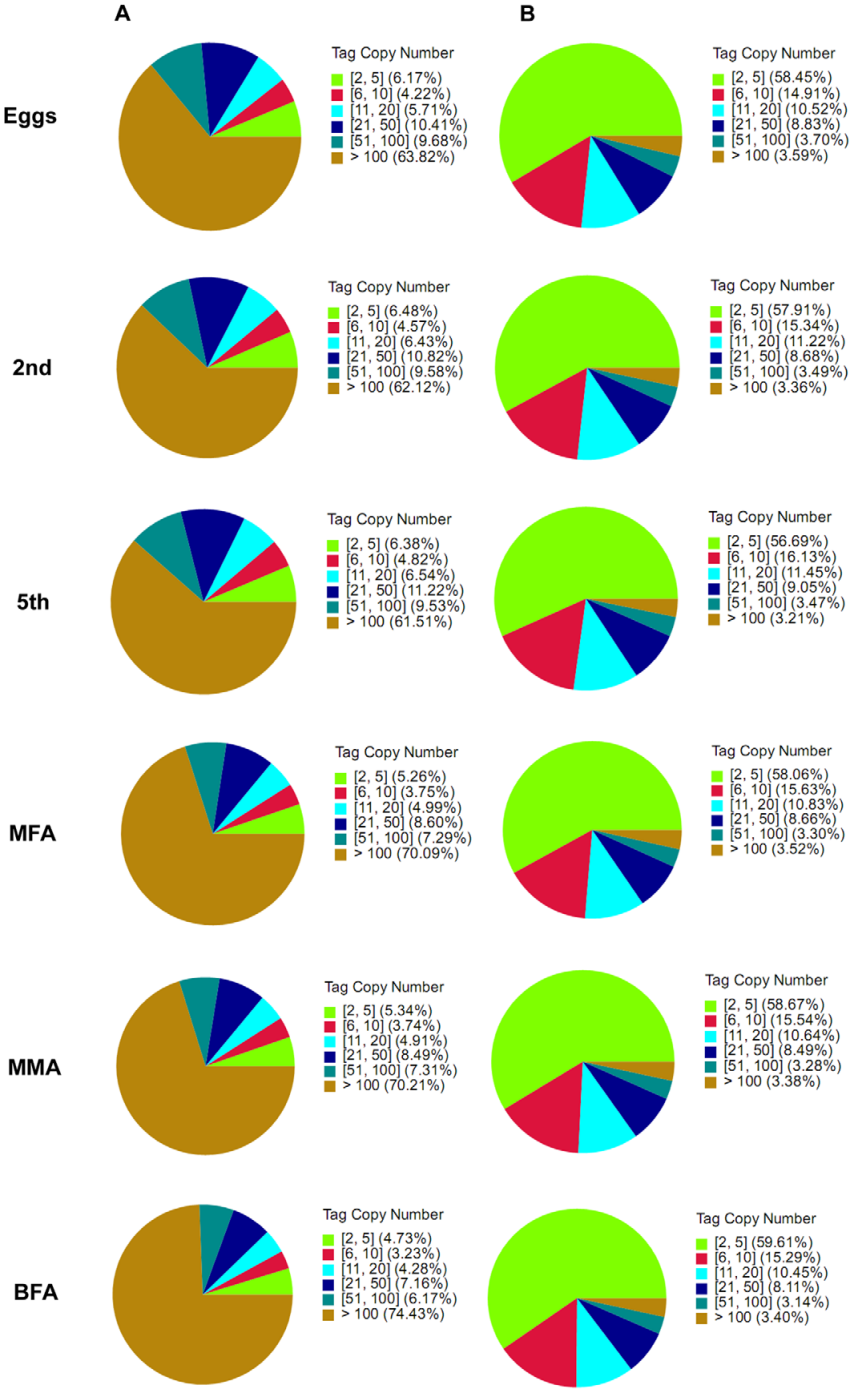
Distribution of total clean tags and distinct clean tags in each sample. The numbers in square brackets indicate the range of copy numbers of each tag category. The data in parentheses indicate the percentage of corresponding tags among the total clean tags and distinct clean tags. (A) Distribution of total clean tags. (B) Distribution of distinct clean tags.

To evaluate the DGE data, we analyzed the distribution of the expression of clean tags (Fig. 5). In each library, the highly expressed genes, *i.e*., those genes with copy numbers of more than 100, showed percentages of greater than 60% among the clean tags, but their distribution of distinct clean tags did not exceed 4%. In contrast, the genes with a low level of expression, *i.e*., those genes with copy numbers of less than five, showed a broad distribution of distinct clean tags in each library.

### Gene expression variations among the different developmental stages

To identify the differentially expressed genes during different developmental stages, the number of clean tags for each gene was calculated, and the genes that were differentially expressed between the two samples were identified using an algorithm developed by Audic *et al* [10].

**Figure 6:**
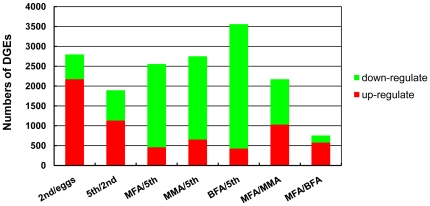
Numbers of DGE unigenes in each comparison. Up-(red) and down-regulated (green) unigenes were quantified. The results of seven comparisons are shown.

The gene expression variations were analyzed between the comparisons of eggs and 2nd instar nymphs, 2nd instar nymphs and 5th instar nymphs, and 5th instar nymphs and adults, respectively. The results revealed 2,798 genes with significantly differential expression levels between the eggs and 2nd instar nymph libraries (Fig. 6). Among them, 2,171 and 627 genes were up-regulated and down-regulated, respectively, in the 2nd instar nymph stage compared to the egg stage. Giving insights into ten of the most differentially up-regulated and ten of the most down-regulated genes, only four of ten up-regulated genes have defined functions, *i.e.*, *fatty acyl-CoA desaturase*, an actin family cytoskeletal gene (*AGAP001622-PA*) and two visual related genes (*rhodopsin* and *opsin-1*), and three of ten down-regulated genes have defined functions, *i.e.*, two energy metabolic related genes (*ATP synthase F0 subunit 6* and *NADH dehydrogenase subunit 5*) and a RNA helicase gene (*DEAD box polypeptide 5*). Additionally, a total of 13 genes among the 20 differentially expressed genes had unknown functions or no annotations (Table S5). According to the GO classification, most of the gene sets demonstrated up-regulated expression in the 2nd instar nymph library, and these genes correlated to metabolic processes, *i.e*., carbohydrate metabolic process, amine metabolic process, oxidation reduction and carboxylic acid metabolic process. Some of the up-regulated gene sets were related to embryo and nymph development, *i.e*., steroid binding, and others participated in cuticle formation, *i.e.*, chitin binding and structural constituents of the cuticle (Table S6). In the KO classification, 53 gene sets were significantly enriched, and most of these genes were up-regulated in the 2nd instar nymph library and correlated to metabolic processes, *i.e*., amino acid metabolism, fatty acid metabolism and carbohydrate metabolism (Table S6).

The comparison between the 2nd instar nymph and 5th instar nymph libraries also revealed significant variations in expression. A total of 1,895 genes, including 1,131 up-regulated and 764 down-regulated genes, were identified in 5th instar nymphs compared to 2nd instar nymphs. Among the ten most up-regulated and ten down-regulated expressed genes (Table S5), four annotated genes were up-regulated in 5th instar nymphs, *U4/U6 small nuclear ribonucleoprotein Prp31* (a pre-mRNA-processing factor 31), *pyridoxamine 5-phosphate oxidase* (a pyridoxine metabolism related gene), *transferrin* (an iron delivery gene) and *Eip55E CG5345-PA* (a ecdysone-induced polypeptide); six functional defined genes were down-regulated in 5th instar nymphs including three mitochondrial genes(*cytochrome c oxidase subunit I*, *cytochrome b* and *AGAP006609-PA*), two actin-related genes (*AGAP001622-PA* and *AGAP006023-PA*) and a cuticle gene. Also, seven genes were blasted without annotation (Table S5). Based on the GO functional classification, almost all of the up-regulated gene sets were involved in processes of carbohydrate metabolism, oxidation reduction and catabolism. The gene sets associated with cuticle formation were also enriched in the 5th instar nymph library. Among gene sets enrichment of KO, significant changes in metabolic pathways were observed, for example, amino acid metabolism and carbohydrate metabolism (Table S7). Meanwhile, we compared the gene expression variations between the 5th instar nymph library and those of three kinds of adult. Most of the genes were down-regulated in the different adult types when compared to the 5th instar nymphs. Common gene sets that were up-regulated in the three adult libraries mainly functioned in peptidase activity, carbohydrate metabolism, and constituents of cuticle (Table S8), according to the GO functional classification.

In total, 755 genes demonstrated significant changes between the BFA and MFA libraries. In the MFA library, 576 and 179 genes were up-regulated and down-regulated, respectively, in comparison with the BFA library (Table S9). A total of 11 genes showed no homology among the ten most differentially up-regulated and ten most down-regulated genes (Table S5). Among these up-regulated genes, four of them located in mitochondrion; two genes (*ferritin subunit* and *sarcalumenin precursor*) were involved in metal ion transport; and a gene (*TcasGA2_TC004721*) product composed of immunoglobulin domain and cytokine receptor domain. Total two functional defined down-regulated genes were predicted to encode vitellogenin and pancreatic lipase related protein 1(PLRP1). Almost all of the gene sets enriched in GO were correlated to respiration and energy metabolism, *i.e.*, the mitochondrion, oxidoreductase activity, oxidation reduction and the generation of precursor metabolites and energy (Table S10). The gene sets enriched in KO were also related to respiration and energy metabolism, *i.e.*, the citrate cycle and oxidative phosphorylation. The PPAR signaling pathway, which is thought to participate in lipid metabolism [11], was up-regulated in MFA (Table S10).

The comparative analysis between the MMA and MFA libraries revealed 2,172 genes with significant expression changes. Among these genes, 1,029 up-regulated and 1,143 down-regulated genes were identified in the MFA compared to the MMA library (Table S11). A total of nine genes showed no homology among the ten most differentially up-regulated and ten most down-regulated expressed genes (Table S5). Among the ten up-regulated genes, three encode ribosomal protein; two genes (*asparagine synthetase* and *l-allo-threonine aldolase*) were involved in amino acid metabolic; and another two genes were *FK506-binding nuclear protein* and *pyridine nucleotide-disulphide oxidoreductase*. Among the ten down-regulated genes, two genes (*GA12219* and *cytosol aminopeptidase*) belong to cytosol aminopeptidase family; a gene (*juvenile hormone esterase*) was involved in juvenile hormone metabolism; and a gene was homologous to gene of *Bacillus halodurans* C-125. In the GO enrichment, most of the gene sets up-regulated in female adults correlated to transcription and translation, *i.e.*, translation, ribonucleoprotein complex biogenesis and ribosome biogenesis. In contrast, the up-regulated gene sets in male adults were related to peptidase activity, carbohydrate metabolism and microtubule dynamics, *i.e.*, hexose metabolic process, glucose metabolic process, serine hydrolase activity, exopeptidase activity, metalloexopeptidase activity and microtubule-based process (Table S12). In the KO enrichment, the gene sets associated with translation were up-regulated in females, such as processes associated with ribosome and spliceosome. In particular, the genes encoding ribosomal proteins had a higher copy number in females compared to males (Table S12). Some gene sets involved in energy metabolic were up-regulated in male, such as citrate cycle, glycolysis/gluconeogenesis and pyruvate metabolism. In particular, components of the insulin signaling pathway showed significant changes between these two libraries.

### Gene expression analysis and qRT-PCR validation

**Figure 7:**
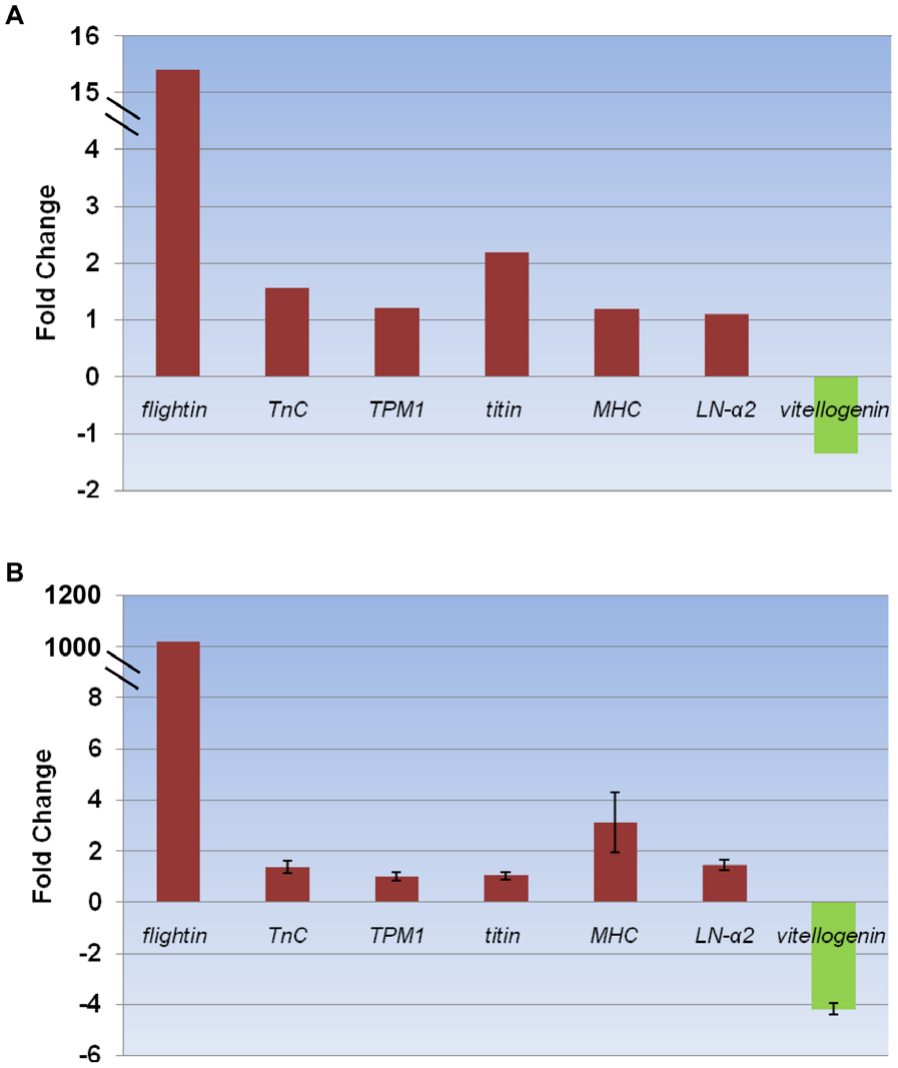
Wing dimorphism-related genes. Red bars represent genes related to muscle cytoskeletal architecture, and green bars represent *vitellogenin*. (A) Gene expression data for DGE analysis. The fold changes of the genes were calculated as the log2 value of each MFA/BFA comparison and are shown on the y-axis. (B) The qRT-PCR analysis of gene expression data. Expression ratios of *flightin*, *TnC*, *TPM1*, *Titin*, *MHC*, *LN-α2* and *vitellogenin* in MFA compared to BFA.

We also focused on the genes related to wing dimorphism. In the MFA and BFA libraries, *vitellogenin* was the most abundantly expressed gene, which demonstrated higher expression levels in BFA compared to MFA. The genes related to muscle cytoskeletal architecture had higher copy numbers in the MFA compared to the BFA library, *i.e*., *flightin*, *TnC*, *TPM1*, *titin*, *myosin heavy chain* (*MHC*) and *laminin-α2* (*LN-α2*) (Table S13; Fig. 7A). The qRT-PCR data for these genes were consistent with the DGE results (Fig. 7B).

**Figure 8:**
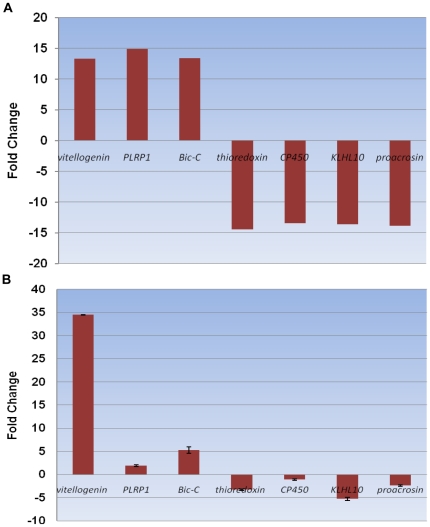
Sex difference-related genes. (A) The fold changes of the genes were calculated as the log2 value of each MFA/BFA comparison and are shown on the y-axis. (B) Gene expression data obtained by qRT-PCR analysis. Expression ratios of seven genes (*vitellogenin*, *PLRP1*, *Bic-C*, *thioredoxin*, *CP450*, *KLHL10* and *proacrosin*) in MFA compared to MMA.

A comparison of the MFA and MMA libraries revealed a high expression level of *vitellogenin* in both libraries, which showed significantly higher expression in the MFA compared to the MMA library. The *PLRP1* and *Bic-C* genes demonstrated significantly higher expression levels in the MFA compared to the MMA library. Some genes related to sex difference showed up-regulated expression levels in the MMA compared to MFA library, such as the genes encoding *thioredoxin*, *CP450*, *KLHL10* and *proacrosin* (Fig. 8A). All of these genes were selected randomly, and the qRT-PCR results were identical to those obtained by DGE expression profiling (Fig. 8B).

## Discussion

BPH is one of the most problematic insect pests of rice in Asia. Although the ecological and physiological mechanisms associated with BPH have been extensively studied, the development modulation, wing dimorphism and sex difference are remaining unclear.

Using transcriptome sequence analysis, it was surprising to find that *T. castaneum* shared the highest similarity with BPH in the BLAST annotation, whereas *A. pisum*, belonging to Hemiptera as BPH, showed a lower best match percentage. These results are not likely due to the availability of more sequence resources of *T. castaneum* compared to *A. pisum* in the NCBI protein database, as the numbers of protein sequences of *T. castaneum*, *P. h. corporis* and *A. pisum* in NCBI Nr database were comparable, containing 27,306, 22,872 and 27,746 protein sequences, respectively. To further confirm this, we downloaded all of BPH midgut related 1,291 ESTs (http://bphest.dna.affrc.go.jp/), and searched the ESTs in library containing all protein sequences of *T. castaneum*, *P. h. corporis* and *A. pisum* in NCBI Nr database using BLSATX with a cut-off e-value of 10^−5^. Eventually, total 746 ESTs provided a BLAST result. Strikingly, a total of 93.7% ESTs showed the best matches with *T. castaneum* followed by *P. h. corporis* (4%), *A. pisum* (2.3%). According to these results, we reasoned that, from a certain aspect, BPH shows the most similarity with *T. castaneum* among these three species. The relationship among these species needs further studies.

To better understand the gene expression information obtained for BPH, we constructed cDNA libraries of different developmental stages, sexes and wing forms to obtain complete transcriptome information. We also created six DEG libraries to analyze the gene expression patterns under different physiological conditions. The mean length reference data (30,987 unigenes) in our study was 841 bp and the N50 was 933 bp, which was much better than sequence quality of EST database (10,200 unigenes with average size 627 bp) [4]. Furthermore, our unigene data have much less redundancy sequences than that of EST data. In the combined 30,987 unigenes, only 16.4% sequence data come from NCBI EST data in terms of the total sizes of the sequences. Our transcriptome and gene expression profiling data greatly enriched the current BPH database and will contribute to research with respect to the identification of novel genes, chemical targets, developmental mechanisms, wing dimorphism and sex difference of BPH. In the developmental stage, most of differentially expressed genes were up-regulated in 2nd instar nymphs compared to eggs. In contrast, most of these genes were down-regulated in adults compared to 5th instar nymphs. In egg and nymph libraries, large amount of genes showed specific expression in these stages, which were likely involved in development differentiations (Table S14).

Wing dimorphism is an important physiological mechanism of BPH, which is considered to be affected by environmental factors, population density, nutrition, juvenile hormones, interspecific interactions and abiotic factors [12], [13], [14], [15]; however, the mechanisms responsible for these processes are remain unclear. Macropterous form might show different trait among adult ages, because they tend to fly just after emergence and they settle on rice plant several days later. In the present study, we identified several gene sets involved in respiration and energy metabolism that showed significantly up-regulated expression levels in MFA compared to BFA (Table S10), which suggested that MFA required more energy than BFA for flight [16]. The PPAR signaling pathway might play a key role in MFA migration. This pathway can regulate lipid metabolism, which provides energy for flying. In human, PPAR pathway plays a role in the metabolically active skeletal muscle [17]. PPAR might be also important for BPH flight muscle activity. Based on the DGE gene expression profiling, some genes associated with muscle cytoskeletal architecture, *i.e*., *flightin*, *TnC*, *TPM1*, *titin*, *MHC* and *LN-α2*, demonstrated higher expression levels in MFA than BFA. The qRT-PCR confirmed the observed up-regulation of these genes, and these results imply that flight muscle may differ between the two wing forms. Among the six genes, *flightin* is the only one exhibiting the dramatically up-regulated level in MFA (Table S14), which is supposed to be vital in flight muscle constitution. Meanwhile, this gene exclusively expressed in 5th instar nymphs except for macropterous adults with low copy numbers (data not shown), suggesting that *flightin* may be closely related to wing form differentiation in nymph stage and may be served as a marker gene for the further studies of the wing form differentiation. Meanwhile, some genes might be also important for development of BFA, *i.e.*, *40S ribosomal protein S20* and *PLRP1* (Table S14 and Table S5). In addition, significantly higher copy numbers of the *vitellogenin* gene were detected in BFA compared to MFA, which suggests that the two female adult forms may possess different reproductive capabilities, BFA is believed to be more fertile than MFA.

With respect to sex difference, the genes encoding *vitellogenin*, *PLRP1* and *Bic-C* demonstrated significantly up-regulated expression levels, whereas the *thioredoxin*, *CP450*, *KLHL10* and *proacrosin* genes showed down-regulated expression levels in MFA compared to MMA, according to DGE profiling. The qRT-PCR results confirmed the observed up-regulation of the *Bic-C* and *PLRP1* genes and the down-regulation of the *CP450*, *KLHL10* and *proacrosin* genes. These genes participate in different pathways; however, they may be involved in sex difference in BPH [18], [19], [20]. Among them, *proacrosin* is proven to locate in acrosomal cap as one of the compounds [21]. According to the results obtained for the gene set enrichments in the present study, BPH female adults appeared to possess more active transcriptional and translational processes than did male adults. In addition, differences in alternative splicing could affect sexual dimorphism at the mRNA level, as most of the genes associated with the spliceosome were up-regulated in MFA [22], [23]. Moreover, some genes are likely not only related to BPH sex difference but also participate in mechanisms related to insecticide resistance, *i.e*., *cytochrome P450* (*CP450*). In *Drosophila*, several *CP450* genes were proved to associate with insecticide resistance [24], [25], while the types of *CP450* in our study has not defined yet. Furthermore, a large number of candidate genes showed significantly different expression but require further characterization.

Although the molecular functions of individual BPH genes and the associated signal transduction pathways remain largely unknown, the present transcriptome analysis provides valuable information regarding BPH development, wing dimorphism and sex difference, which could facilitate further investigations of the detailed physiological mechanisms of BPH pest insects.

## Supporting Information

Table S1 Primers used in qRT-PCR for validation of differentially expressed genes.
(0.02 MB XLS)

Table S2 Top hits obtained by BLASTX for the unigenes. BLASTX against the nr protein database was used with a cutoff E value < 1e-5.
(2.83 MB XLS)

Table S3 GO annotation of unigenes. We combined the GO annotation provided by Blast2Go and InterProScan.
(2.59 MB XLS)

Table S4 KO annotation of unigenes
(1.65 MB XLS)

Table S5 Top ten differentially expressed genes in each library of comparisons.
(0.16 MB DOC)

Table S6 Gene set enrichment analysis comparing 2nd instar nymphs and eggs.
(0.04 MB XLS)

Table S7 Gene set enrichment analysis comparing 5th instar nymphs and 2nd instar nymphs.
(0.03 MB XLS)

Table S8 Gene set enrichment analysis comparing MFA, MMA and BFA with 5th instar nymphs.
(0.05 MB XLS)

Table S9 Differentially expressed genes between MFA and BFA.
(0.17 MB XLS)

Table S10 Gene set enrichment analysis comparing MFA and BFA.
(0.03 MB XLS)

Table S11 Differentially expressed genes between MFA and MMA.
(0.45 MB XLS)

Table S12 Gene set enrichment analysis comparing MFA and MMA.
(0.03 MB XLS)

Table S13 Muscle cytoskeletal architecture-related genes.
(0.03 MB XLS)

Table S14 Top ten specific expressed genes in each library comparison.
(0.04 MB XLS)
