# Supplementary material for: Transcriptome Analysis of the Brown Planthopper Nilaparvata lugens
Source: PLoS One. 2010 Dec 6;5(12):e14233. doi: 10.1371/journal.pone.0014233 (PMC2997790; doi:10.1371/journal.pone.0014233)
Supplement: Table S5 — Top ten differentially expressed genes in each library of comparisons. (0.16 MB DOC) [file pone.0014233.s005.doc]

**Table S5. Top ten differentially expressed genes in each library of comparisons.**

| **Comparison** | **Unigene**  **ID** | **Hit Number** | **Discription** | **FDR** | **Fold Change** |
| --- | --- | --- | --- | --- | --- |
| **2nd instar /eggs** | **NL30534^a^** | **-** | **-** | **0** | **16.34** |
|  | **NL00492^a^** | **gi\|23393789\|gb\|AAN31393.1\|** | **fatty acyl-CoA desaturase [*Musca domestica*]** | **0** | **16.13** |
|  | **NL06133^a^** | **gi\|118794438\|ref\|XP_321477.2\|** | **AGAP001622-PA [*Anopheles gambiae str.* PEST]** | **0** | **15.21** |
|  | **NL21346^a^** | **-** | **-** | **0** | **14.86** |
|  | **NL14843^a^** | **gi\|51557683\|gb\|AAU06481.1\|** | **rhodopsin [*Culicoides sonorensis*]** | **0** | **14.68** |
|  | **NL09563^a^** | **gi\|194753464\|ref\|XP_001959032.1\|** | **GF12674 [*Drosophila ananassae*]** | **0** | **14.39** |
|  | **NL00840^a^** | **gi\|193636528\|ref\|XP_001947537.1\|** | **PREDICTED: similar to soldier-specific protein-1 [*Acyrthosiphon pisum*]** | **0** | **13.91** |
|  | **NL19783^a^** | **gi\|170052387\|ref\|XP_001862198.1\|** | **opsin-1 [*Culex quinquefasciatus*]** | **0** | **13.89** |
|  | **NL27165^a^** | **-** | **-** | **0** | **13.86** |
|  | **NL12501^a^** | **-** | **-** | **0** | **13.79** |
|  | **NL00339^b^** | **gi\|228015381\|ref\|YP_002836171.1\|** | **ATP synthase F0 subunit 6 [*Geisha distinctissima*]** | **0** | **-3.29** |
|  | **NL08961^b^** | **gi\|240266564\|ref\|YP_002970935.1\|** | **NADH dehydrogenase subunit 5 [*Lycorma delicatula*]** | **0** | **-2.57** |
|  | **NL22255^b^** | **-** | **-** | **0** | **-2.39** |
|  | **NL01131^b^** | **gi\|270013489\|gb\|EFA09937.1\|** | **hypothetical protein TcasGA2_TC012090 [*Tribolium castaneum*]** | **0** | **-2.27** |
|  | **NL09289^b^** | **-** | **-** | **0** | **-1.98** |
|  | **NL00317^b^** | **-** | **-** | **0** | **-1.52** |
|  | **NL27253^b^** | **-** | **-** | **0** | **-1.49** |
|  | **NL04919^b^** | **gi\|156546614\|ref\|XP_001602649.1\|** | **PREDICTED: similar to ENSANGP00000007330 [*Nasonia vitripennis*]** | **3.38E-297** | **-2.32** |
|  | **NL24110^b^** | **-** | **-** | **5.02E-287** | **-2.74** |
|  | **NL00160^b^** | **gi\|189242418\|ref\|XP_001811120.1\|** | **PREDICTED: similar to DEAD box polypeptide 5 [*Tribolium castaneum*]** | **2.27E-276** | **-1.46** |
| **5th instar**  **/2nd instar** | **NL16490^a^** | **gi|221135713|ref|XP_002170981.1|** | **PREDICTED: similar to predicted protein, partial [*Hydra magnipapillata*]** | **0** | **3.06** |
|  | **NL05893^a^** | **gi\|270003644\|gb\|EFA00092.1\|** | **hypothetical protein TcasGA2_TC002907 [*Tribolium castaneum*]** | **0** | **3.05** |
|  | **NL24889^a^** | **-** | **-** | **0** | **2.81** |
|  | **NL16226^a^** | **gi\|242022928\|ref\|XP_002431889.1\|** | **U4/U6 small nuclear ribonucleoprotein Prp31, putative [*Pediculus humanus corporis*]** | **0** | **2.31** |
|  | **NL02110^a^** | **gi\|193704781\|ref\|XP_001947706.1\|** | **PREDICTED: similar to Putative pyridoxamine 5-phosphate oxidase, partial [*Acyrthosiphon pisum*]** | **0** | **2.14** |
|  | **NL00685^a^** | **gi\|156891049\|gb\|ABU96701.1\|** | **transferrin [*Rhodnius prolixus*]** | **0** | **2.13** |
|  | **NL00262^a^** | **-** | **-** | **0** | **2.02** |
|  | **NL24371^a^** | **-** | **-** | **0** | **1.80** |
|  | **NL21030^a^** | **gi\|193610981\|ref\|XP_001949651.1\|** | **PREDICTED: similar to Eip55E CG5345-PA, partial [*Acyrthosiphon pisum*]** | **0** | **1.76** |
|  | **NL29468^a^** | **-** | **-** | **0** | **1.73** |
|  | **NL14053^b^** | **gi\|225347350\|ref\|YP_002640588.2\|** | **cytochrome c oxidase subunit I [*Hydroscapha granulum*]** | **0** | **-16.05** |
|  | **NL16821^b^** | **gi\|157326172\|ref\|YP_001468385.1\|** | **cytochrome b [*Culicoides arakawae*]** | **0** | **-15.54** |
|  | **NL04454^b^** | **gi\|193636635\|ref\|XP_001950829.1\|** | **PREDICTED: similar to conserved hypothetical protein [*Acyrthosiphon pisum*]** | **0** | **-15.45** |
|  | **NL06133^b^** | **gi\|118794438\|ref\|XP_321477.2\|** | **AGAP001622-PA [*Anopheles gambiae str.* PEST]** | **0** | **-15.21** |
|  | **NL15564^b^** | **gi\|158296157\|ref\|XP_316638.4\|** | **AGAP006609-PA [*Anopheles gambiae str.* PEST]** | **0** | **-6.60** |
|  | **NL09289^b^** | **-** | **-** | **0** | **-4.35** |
|  | **NL07084^b^** | **-** | **-** | **0** | **-3.68** |
|  | **NL26619^b^** | **-** | **-** | **0** | 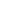**-3.42** |
|  | **NL00928^b^** | **gi\|158295182\|ref\|XP_316061.3\|** | **AGAP006023-PA [*Anopheles gambiae str.* PEST]** | **0** | **-3.27** |
|  | **NL16299^b^** | **gi\|223671248\|tpd\|FAA00576.1\|** | **TPA: putative cuticle protein [*Bombyx mori*]** | **0** | **-3.08** |
| **MFA**  **/BFA** | **NL00111^a^** | **gi|6688881|emb|CAB65310.1|** | **ferritin subunit (non-glycosylated) [*Nilaparvata lugens*]** | **0** | **3.28** |
|  | **NL01435^a^** | **gi\|189239144\|ref\|XP_971313.2\|** | **PREDICTED: similar to dihydrolipoamide succinyltransferase component of 2-oxoglutarate dehydrogenase [*Tribolium castaneum*]** | **0** | **2.81** |
|  | **NL02753^a^** | **gi\|229892296\|ref\|NP_001153501.1\|** | **oxoglutarate (alpha-ketoglutarate) dehydrogenase (lipoamide) [*Nasonia vitripennis*]** | **0** | **2.73** |
|  | **NL01739^a^** | **gi\|270007569\|gb\|EFA04017.1\|** | **hypothetical protein TcasGA2_TC014166 [*Tribolium castaneum*]** | **0** | **2.73** |
|  | **NL01029^a^** | **gi\|263173502\|gb\|ACY69956.1\|** | **mitochondrial phosphate carrier protein [*Cimex lectularius*]** | **0** | **2.49** |
|  | **NL00578^a^** | **-** | **-** | **0** | **2.28** |
|  | **NL00627^a^** | **gi\|270014673\|gb\|EFA11121.1\|** | **hypothetical protein TcasGA2_TC004721 [*Tribolium castaneum*]** | **0** | **2.20** |
|  | **NL22995^a^** | **-** | **-** | **0** | **2.17** |
|  | **NL00980^a^** | **gi\|242006712\|ref\|XP_002424191.1\|** | **Sarcalumenin precursor, putative [*Pediculus humanus corporis*]** | **0** | **2.04** |
|  | **NL29164^a^** | **-** | **-** | **0** | **2.04** |
|  | **NL00643^b^** | **-** | **-** | **0** | **-2.72** |
|  | **NL30565^b^** | **-** | **-** | **0** | **-2.57** |
|  | **NL00463^b^** | **-** | **-** | **0** | **-2.54** |
|  | **NL05048^b^** | **-** | **-** | **0** | **-2.34** |
|  | **NL01214^b^** | **-** | **-** | **0** | **-2.28** |
|  | **NL23336^b^** | **-** | **-** | **0** | **-2.13** |
|  | **NL05450^b^** | **-** | **-** | **0** | **-2.11** |
|  | **NL00460^b^** | **-** | **-** | **0** | **-2.06** |
|  | **NL00012^b^** | **gi\|154799937\|dbj\|BAF75351.1\|** | **vitellogenin [*Nilaparvata lugens*]** | **0** | **-1.97** |
|  | **NL01993^b^** | **gi\|193587079\|ref\|XP_001947773.1\|** | **PREDICTED: similar to pancreatic lipase related protein 1 [*Acyrthosiphon pisum*]** | **0** | **-1.93** |
| **MFA**  **/MMA** | **NL22066^a^** | **-** | **-** | **0** | **5.42** |
|  | **NL01090^a^** | **gi\|242020998\|ref\|XP_002430934.1\|** | **asparagine synthetase, putative [*Pediculus humanus corporis*]** | **0** | **3.62** |
|  | **NL00504^a^** | **gi\|110777718\|ref\|XP_001121759.1\|** | **PREDICTED: similar to 39 kDa FK506-binding nuclear protein (Peptidyl-prolyl cis-trans isomerase) (PPIase) (Rotamase) [*Apis mellifera*]** | **0** | **3.14** |
|  | **NL00414^a^** | **gi\|193693000\|ref\|XP_001948416.1\|** | **PREDICTED: similar to l-allo-threonine aldolase, partial [*Acyrthosiphon pisum*]** | **0** | **3.08** |
|  | **NL23063^a^** | **-** | **-** | **0** | **3.01** |
|  | **NL00345^a^** | **gi\|66558956\|ref\|XP_392812.2\|** | **PREDICTED: similar to Ribosomal protein L23 CG3661-PA [*Apis mellifera*]** | **0** | **2.77** |
|  | **NL00500^a^** | **gi\|50344488\|emb\|CAH04330.1\|** | **S14e ribosomal protein [*Dascillus cervinus*]** | **0** | **2.75** |
|  | **NL02005^a^** | **gi\|193659748\|ref\|XP_001946449.1\|** | **PREDICTED: similar to AGAP010399-PA [*Acyrthosiphon pisum*]** | **0** | **2.66** |
|  | **NL00982^a^** | **-** | **-** | **0** | **2.63** |
|  | **NL00290^a^** | **gi\|90820018\|gb\|ABD98766.1\|** | **putative ribosomal protein L27Ae [*Graphocephala atropunctata*]** | **0** | **2.60** |
|  | **NL01844^b^** | **-** | **-** | **0** | **-17.00** |
|  | **NL22094^b^** | **-** | **-** | **0** | **-16.71** |
|  | **NL01015^b^** | **gi\|170317966\|gb\|ACB14344.1\|** | **juvenile hormone esterase [*Nilaparvata lugens*]** | **0** | **-15.87** |
|  | **NL00642^b^** | **gi\|15613784\|ref\|NP_242087.1\|** | **hypothetical protein BH1221 [*Bacillus halodurans* C-125]** | **0** | **-15.87** |
|  | **NL02380^b^** | **gi\|198460106\|ref\|XP_001361611.2\|** | **GA12219 [*Drosophila pseudoobscura pseudoobscura*]** | **0** | **-15.78** |
|  | **NL01144^b^** | **-** | **-** | **0** | **-15.69** |
|  | **NL02327^b^** | **gi\|170046819\|ref\|XP_001850946.1\|** | **cytosol aminopeptidase [*Culex quinquefasciatus*]** | **0** | **-15.50** |
|  | **NL01912^b^** | **-** | **-** | **0** | **-15.46** |
|  | **NL01255^b^** | **-** | **-** | **0** | **-15.40** |
|  | **NL22130^b^** | **-** | **-** | **0** | **-15.31** |

**The corner marks of gene ID: ‘a’ represents up-regulated genes and ‘b’ represents down-regulated genes. The expression fold changes were performed with log 2 ratio.**
